# Actinomycosis mimicking malignancy

**DOI:** 10.1590/0037-8682-0180-2024

**Published:** 2024-07-29

**Authors:** Buğra Kerget, Alperen Aksakal, Sevilay Özmen

**Affiliations:** 1 Ataturk University School of Medicine, Department of Pulmonary Diseases, 25240, Yakutiye, Erzurum, Turkey.; 2 Ataturk University School of Medicine, Department of Pathology, 25240, Yakutiye, Erzurum, Turkey.

**FIGURE 1:**
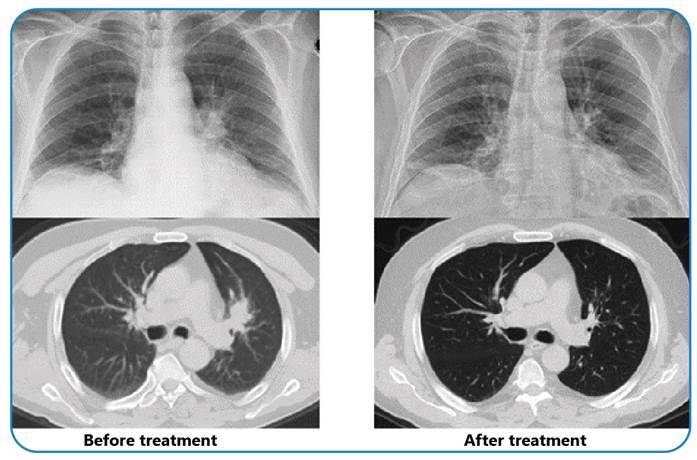
Comparison of radiological findings before and after treatment.

**FIGURE 2:**
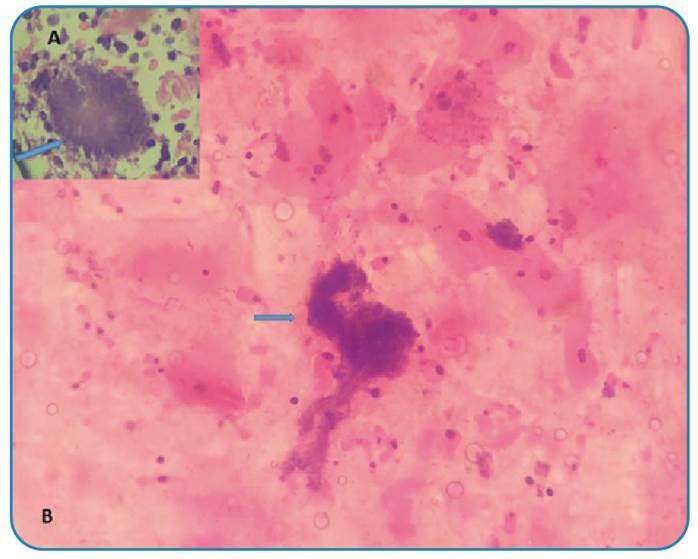
Basophilic filamentous bacterial aggregates stained with hematoxylin and eosin dye **(A, B)**.

A 70-year-old male patient was admitted to the emergency department with persistent cough, hemoptysis, and back pain that had lasted for three weeks. Although his physical examination in the emergency department revealed no significant findings, a chest radiograph displayed a consolidated area with spicular extension in the left hilar region. Subsequent thoracic computed tomography (CT) showed a mass lesion measuring 38x25x36 mm with a standard uptake value (SUV) of 7.44 adjacent to the hilar region (Figure 1). Suspecting lung malignancy, the medical team performed endobronchial ultrasonography (EBUS), which identified a heterogeneous hypoechoic intramural lesion on the anterior wall of the left upper lobe entrance. Cytological samples were taken from the lesion twice, which tested negative for malignancy. However, they revealed bacterial clusters consistent with Actinomyces (Figure 2). The patient was initially treated with 4 x 2 g of intravenous ampicillin for eight weeks. A follow-up CT scan showed complete regression of the mass in the upper lobe (Figure 1). Treatment continued with amoxicillin plus clavulanic acid, administered orally at a dosage of 3 x 1 g for an additional eight weeks, during which he was closely monitored. Pulmonary actinomycosis is known to mimic a range of lung pathologies, from benign infections to metastatic tumors^1^, and 25% of thoracic actinomycosis cases are initially misdiagnosed as malignancy^2^. The patient achieved complete recovery with appropriate treatment over a sufficient duration.
